# The Association of Diabetic Cheiroarthropathy With Microvascular Complications of Type 2 Diabetes Mellitus: A Cross-Sectional Study

**DOI:** 10.7759/cureus.36701

**Published:** 2023-03-26

**Authors:** Athul Paul, Kothai Gnanamoorthy

**Affiliations:** 1 General Medicine, Sri Ramasamy Memorial (SRM) Medical College Hospital and Research Centre, Potheri, IND

**Keywords:** diabetic retinopathy, diabetic nephropathy, diabetic neuropathy, advanced glycation end products, diabetes cheiroarthropathy

## Abstract

Introduction

Diabetic cheiroarthropathy (DCA), also known as the syndrome of limited joint mobility (LJM), is among the most underdiagnosed complications of diabetes mellitus (DM). Although not severe, it can hamper the day-to-day activities of the patient and significantly reduce the quality of life. It is hypothesized to be due to increased glycation of collagen around joints. The objective of our study was to examine the association of diabetic cheiroarthropathy with microvascular complications of type 2 diabetes mellitus.

Methods

The study was conducted on 251 previously diagnosed cases of type 2 DM. Patients with previous contractures due to any other cause, who are diagnosed cases of rheumatoid arthritis and scleroderma, and other risk factors such as cardiac or renal disease were excluded from the study. All subjects were subjected to a detailed clinical history including a past history, thorough physical examination, prayer test, tabletop sign, and passive extension of fingers. Patients who are diagnosed with diabetic cheiroarthropathy were then screened for microalbuminuria, fundus examination, and monofilament test and clinical examination to look for the presence of microvascular complications.

Results

Out of the 251 patients, 46 (18.3%) were found to have diabetic cheiroarthropathy. Fifteen (34.9%) cheiroarthropathy patients had neuropathy compared to 14.9% without diabetic cheiroarthropathy, which was statistically significant. We found that there was an increased incidence of diabetic neuropathy in subjects with cheiroarthropathy. Thirty (35.7%) patients with diabetic cheiroarthropathy had diabetic retinopathy compared to 9.6% without diabetic cheiroarthropathy. Twenty-six (26.8%) patients with diabetic cheiroarthropathy had diabetic nephropathy compared to 13% without diabetic cheiroarthropathy. We identified from our study that patients with diabetic cheiroarthropathy had an increased risk of developing microvascular complications.

Conclusion

There is an increased prevalence of diabetic nephropathy, diabetic neuropathy, and diabetic retinopathy in patients with diabetic cheiroarthropathy. The presence of diabetic cheiroarthropathy hence warrants better control of the patient’s glycemic status to prevent further deterioration of diabetes-related complications.

## Introduction

Diabetic cheiroarthropathy (DCA), also known as diabetic stiff hand or syndrome of limited joint mobility (LJM), is one of the musculoskeletal disorders that is an underdiagnosed complication of diabetes mellitus (DM) compared to the other widely studied microvascular and macrovascular complications of diabetes mellitus. Diabetic cheiroarthropathy is usually characterized by painless limited extension of the proximal metacarpophalangeal joints and/or interphalangeal joints with spontaneous flexion of the fingers [1]. Typically, asymptomatic contractures start in the distal and proximal interphalangeal joints and progress proximally to affect the metacarpophalangeal joints [2]. The larger joints, such as the elbows, shoulders, knees, and axial skeleton, may gradually become more affected by joint contractures beyond the hands [3].

Collagen that has undergone nonenzymatic glycation becomes excessively cross-linked and resistant to breakdown, leading to an increased buildup of collagen in joints. It is hypothesized that the aberrant collagen deposition in the connective tissue around joints causes the stiffening and accompanying skin changes of restricted joint mobility [4-6].

Physical examination and the elimination of underlying rheumatic disorders, particularly scleroderma and other autoimmune diseases, are used to diagnose diabetic cheiroarthropathy [2].

The diagnosis of restricted joint mobility in the hands is made using two straightforward examinations. The capacity to flatten the hands together as in prayer is tested by the “prayer sign,” which makes contractures in the metacarpophalangeal, proximal interphalangeal, and distal interphalangeal joints easier to spot. The “tabletop test” evaluates the hand’s capacity to flatten on a table’s surface, making it easier to spot contractures in the metacarpophalangeal joints [7]. If this turns out to be impossible, it indicates that there are finger flexion contractures, and the tabletop sign is regarded as positive [7].

When a patient has diabetes, it might be challenging to distinguish DCA from other joint problems. Diabetes patients are more likely than the general population to suffer from certain musculoskeletal diseases, such as Dupuytren’s disease, flexor tenosynovitis, plantar fasciitis, periarthritis shoulder, Charcot’s joint, and carpal tunnel syndrome [7].

Studies have shown that diabetic cheiroarthropathy is directly related to the duration of diabetes mellitus [8-10]. Hill et al. found in their study that cheiroarthropathy appears to be linked to the prolonged duration of diabetes and the existence of microvascular problems [11].

Microvascular complications

Compared to patients without DCA, diabetic patients diagnosed with DCA had a higher risk of proliferative retinopathy, nephropathy, and neuropathy [10,12].

Garg et al. demonstrated an increased prevalence of diabetic retinopathy and microalbuminuria in a cross-sectional study of 357 subjects [13]. In a study done in the Nigerian population, diabetic patients with limited joint mobility had a greater prevalence of cataracts and background retinopathy compared to patients without limited joint mobility [14]. Regardless of glycemic management, the development of DCA was linked to a higher incidence of microalbuminuria [15].

Males with restricted joint motion had higher rates of proteinuria, retinopathy, and hypertension than those with normal joint motion in a cross-sectional study of people with type 1 diabetes [11]. A 15-year prospective study of 37 individuals with type 1 diabetes found that those with microalbuminuria experienced a larger loss in joint mobility than those without it [16].

## Materials and methods

We studied 251 patients with established type 2 diabetes (females and males), aged 18-65 years, attending our hospital either as inpatients or outpatients. Patients with preexisting hand disorders, rheumatoid arthritis, scleroderma, and hand osteoarthritis, patients with deformities of the hand with trauma and surgery and previous contractures, and patients with medical disorders such as end-stage renal diseases, chronic liver diseases, or malignancies were excluded from the study. All subjects gave their informed consent for examination and laboratory studies. The study was approved by the Institutional Ethics Committee of Sri Ramasamy Memorial (SRM) Medical College Hospital and Research Centre (approval number: 2355/IEC/2021).

**Figure 1:**
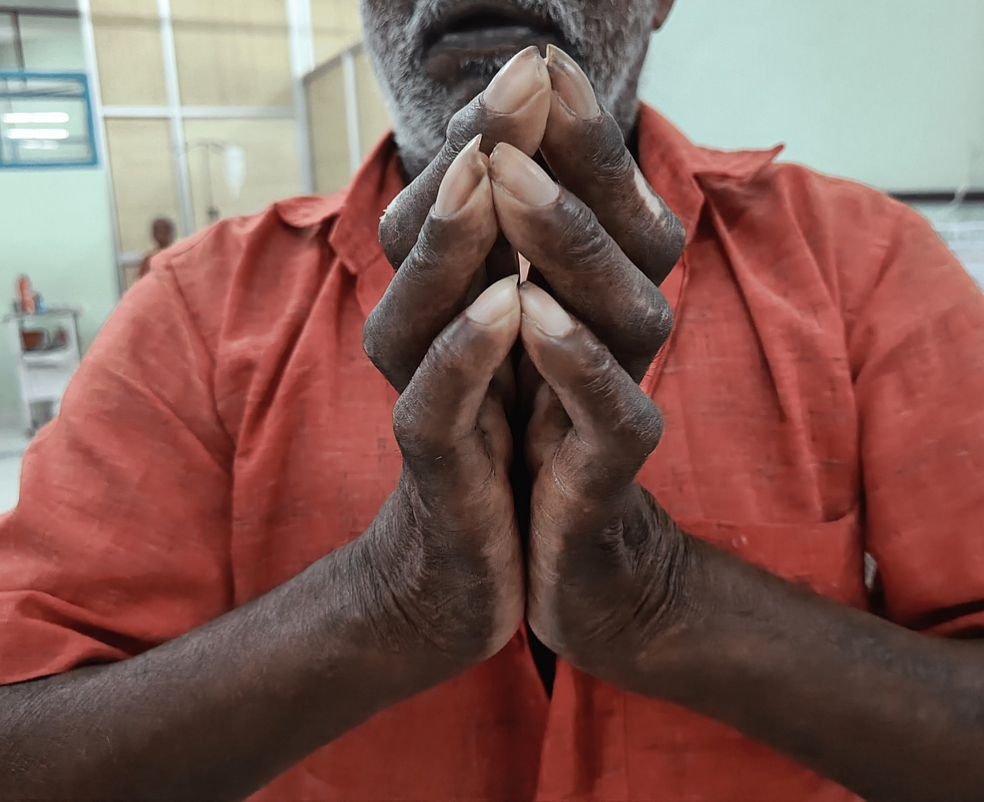
Positive prayer sign in diabetic cheiroarthropathy

Diabetic cheiroarthropathy was assessed by the same examiner for the prayer sign and tabletop sign. In a praying position with their fingers spread and their wrists flexed, patients were instructed to approximate the palmar surfaces of their fingers. No cheiroarthropathy was defined as findings that were ambiguous, unilateral, or merely a sense of resistance without constraints. LJM was the designation for any joint that failed to make contact (Figure 1).

**Figure 2:**
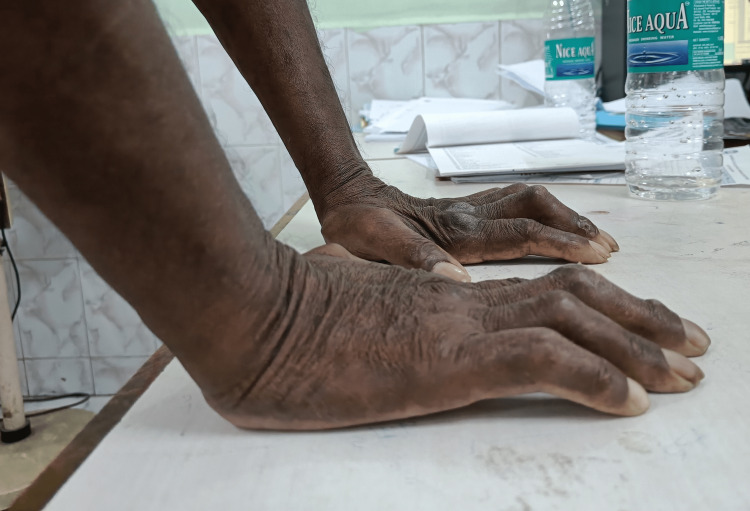
Positive tabletop sign in diabetic cheiroarthropathy

Similarly, the inability to approximate the palmar surface of the hand against a flat surface, i.e., the tabletop, was taken as a positive tabletop sign (Figure 2).

Patients who tested positive for both signs underwent further investigation with antinuclear antibody (ANA) and rheumatoid factor (RF) to rule out the presence of rheumatoid arthritis and systemic sclerosis. The remaining patients were considered to have diabetic cheiroarthropathy.

The patients were then categorized according to the presence or absence of neuropathy, nephropathy, and retinopathy. Nephropathy was defined according to Kidney Disease: Improving Global Outcomes (KDIGO) 2012 guidelines as either one of the following: urine albumin-to-creatinine ratio of more than 30 mg/g or glomerular filtration rate (GFR) of less than 60 mL/minute/1.73 m^2^ or albumin excretion rate of more than 30 mg/24 hours for more than three months [17]. Retinopathy was considered to be present if it was noted in any form by the ophthalmologist during a standardized examination of the fundus through dilated pupils. Neuropathy was screened using monofilament and clinical examination.

## Results

**Figure 3:**
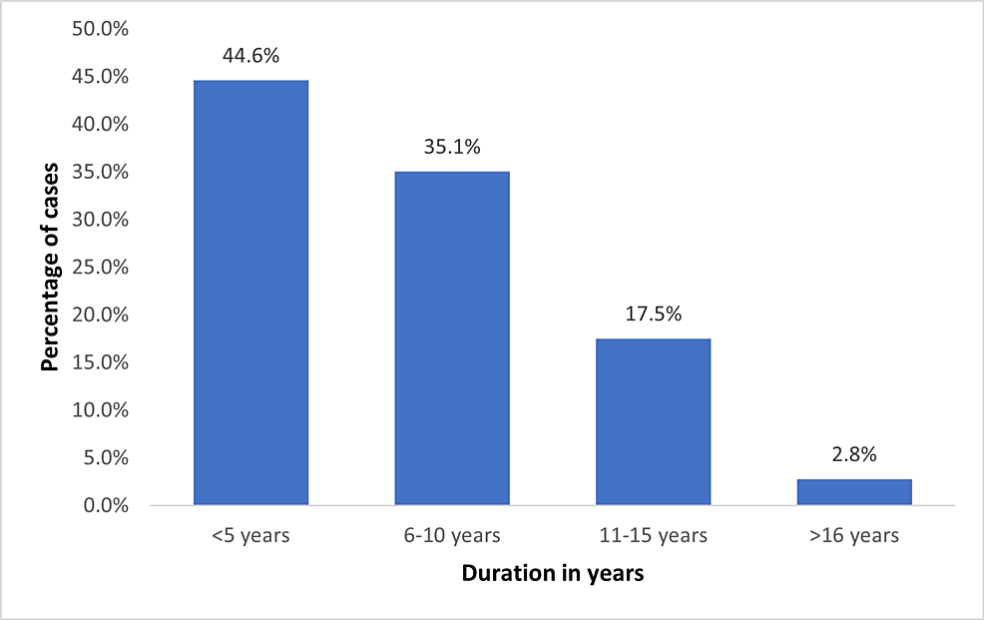
Distribution of the duration of diabetes

A total of 251 subjects were recruited for the study who met the inclusion and exclusion criteria. Among 251 patients, the maximum age group was between 41 and 50, 87 (34.7%), and 51-60 (33.1%). There were 44 (17.5%) patients in the age group under 40 years and 37 (14.7%) in the age group of more than 61 years. Out of the 251 patients, 46 (18.3%) were found to have diabetic cheiroarthropathy. In our study, 24.1% of patients ages 51-60 years, 35.1% of patients over 60 years, and 10.3% of patients ages 41-50 years had diabetic cheiroarthropathy. In analyzing the duration of diabetes in the patients, 8% of patients with diabetes in our study had cheiroarthropathy for a duration of fewer than five years, 26.1% had cheiroarthropathy for a duration of 6-10 years, and 29.5% had cheiroarthropathy for a duration of 11-15 years (Table 1, Figure 3).

**Table 1:** Crosstabulation of diabetes duration with cheiroarthropathy DM: diabetes mellitus

			Cheiroarthropathy		Total	P value
			No	Yes		
Duration of type 2 DM	<5 years	Count	103	9	112	0.002
		% within the duration of type 2 DM	92%	8%	100%	
	6-10 years	Count	65	23	88	
		% within the duration of type 2 DM	73.9%	26.1%	100%	
	11-15 years	Count	31	13	44	
		% within the duration of type 2 DM	70.5%	29.5%	100%	
	>16 years	Count	6	1	7	
		% within the duration of type 2 DM	85.7%	14.3%	100%	
Total		Count	205	46	251	
		% within the duration of type 2 DM	81.7%	18.3%	100%	

Diabetic neuropathy and its association with diabetic cheiroarthropathy

In our study, comparing diabetic neuropathy and cheiroarthropathy, 34.9% of cheiroarthropathy patients had neuropathy compared to 14.9% without cheiroarthropathy.

Diabetic retinopathy and its association with diabetic cheiroarthropathy

Comparing diabetic retinopathy and diabetic cheiroarthropathy, 35.7% of patients with diabetic cheiroarthropathy were found to have diabetic retinopathy compared to 9.6% of patients with no diabetic cheiroarthropathy.

Diabetic nephropathy and its association with diabetic cheiroarthropathy

**Figure 4:**
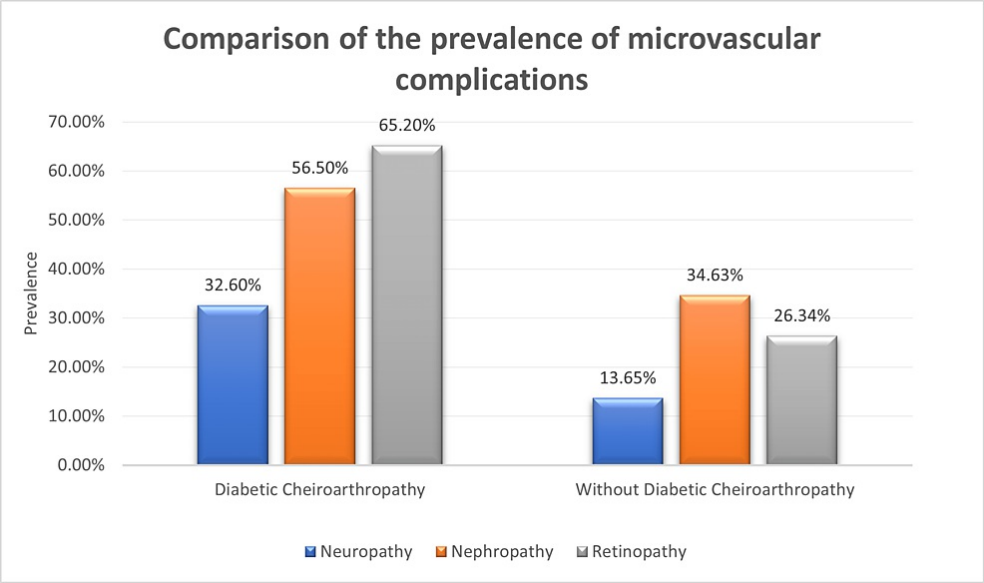
Comparison of the prevalence of microvascular complications between patients with and without diabetic cheiroarthropathy

Comparing diabetic nephropathy and diabetic cheiroarthropathy, 26 (26.8%) of patients with cheiroarthropathy had nephropathy compared to 13% without DCA (Figure 4).

## Discussion

Out of the 251 patients studied, 46 (18.3%) were found to have diabetic cheiroarthropathy. The prevalence of DCA varies from 8% to 58% [2,12,18]. The population being examined, the definition being employed, and how joint mobility is being assessed all play a significant role in this variability. Care must be made to distinguish between restricted joint mobility in the hands and other coexisting diabetic hand issues such as Dupuytren’s contracture, flexor tenosynovitis, and trigger finger [2]. According to a study by Ravindran Rajendran et al. [19], diabetic patients had a 28.5% prevalence of cheiroarthropathy. A study conducted by Ray et al. revealed that 29% of people had DCA [20]. In a different study conducted by Agrawal et al., 22.6% of diabetic patients had cheiroarthropathy [21]. In a recent study done in Kozhikode, India, in 2019, 21.7% of patients who had type 2 diabetes mellitus had limited joint mobility syndrome [22].

In our research, we discovered that the prevalence of cheiroarthropathy increases with increasing age. Twenty (24.1%) patients between ages 51-60 years had cheiroarthropathy, 13 (35.1%) who are more than 60 years had cheiroarthropathy, and nine (10.3%) patients between ages 41 and 50 had cheiroarthropathy. This was in accordance with previous studies that showed that increasing age is an important factor in the prevalence of DCA [10,22].

While comparing the duration of diabetes mellitus and cheiroarthropathy, 23 (26.1%) subjects had a duration of 6-10 years of diabetes, and 13 (29.5%) patients had a duration of 11-15 years, which is a statistically significant difference. Hence, we found that patients with a longer duration of diabetes mellitus had a higher probability of developing cheiroarthropathy. This was in accordance with the findings in the previous studies that showed a positive correlation between the duration of diabetes and the occurrence of diabetic cheiroarthropathy [8-10]. The researchers also discovered that patients with prepubertal onset of DM had more severe limitations in joint mobility than those whose diagnoses were established after puberty. They offered the explanation that during the pubertal development spurt, hyperglycemia causes the laying down of significant volumes of highly glycated collagens [23]. A longitudinal cohort research that found a higher incidence of LJM with longer DM duration and with puberty corroborated the finding [24].

Compared to the frequency two decades ago, LJM is now less common, according to two studies, one in children [25] and the other in adults [26]. The development of more intense and aggressive glycemic management measures over the intervening period is credited with this transformation.

Association with microvascular complication

In 1981, Rosenbloom first discovered a significant correlation between the incidence of microvascular illness in type 1 diabetes mellitus and the degree of joint restriction [27]. They found that patients with DCA had a threefold increased incidence of clinically evident microvascular damage. They came to the conclusion that people with limited joint mobility are more likely to develop early-onset microvasculopathy. This conclusion has been validated by further research [5,28].

**Figure 5:**
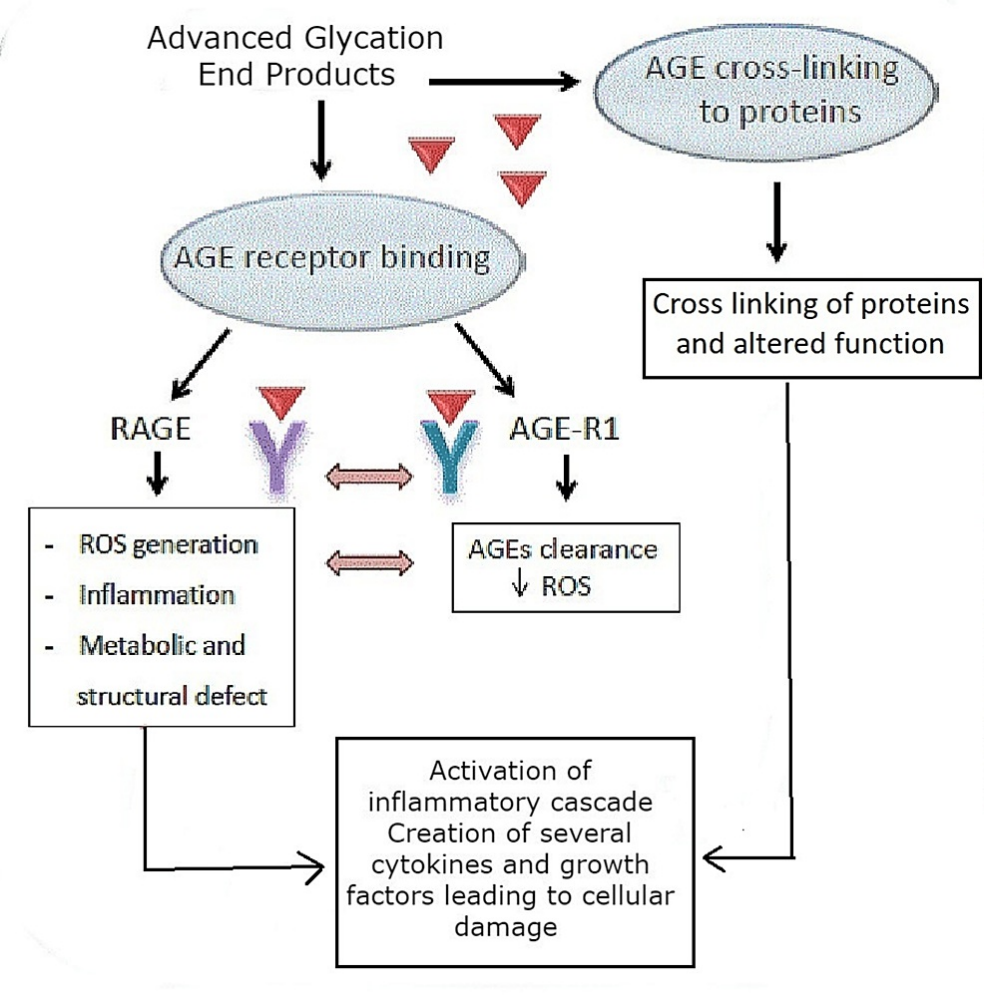
Advanced glycation end product pathway in diabetic cheiroarthropathy AGE: advanced glycation end product, RAGE: receptor for advanced glycation end product, ROS: reactive oxygen species

Nonenzymatic glycation of collagen, similar to that of glycated hemoglobin, is one effect of persistent hyperglycemia. In DM patients, this glycosylation leads to abnormally cross-linked collagens that are particularly resistant to mechanical and enzymatic destruction and collagen buildup [2,23,24]. Additionally, the glycated collagen may trap potentially damaging non-glycated proteins, including albumin, immunoglobulins, and coagulation proteins, and this may exacerbate the formation of the extracellular matrix [29]. Advanced glycation end products (AGEs) are harmful products that develop nonenzymatically under conditions of oxidative stress and hyperglycemia. This causes an increase in the level of reactive oxygen species (ROS) that activates an inflammatory cascade that results in the creation of several cytokines and growth factors that cause cellular damage (Figure 5) [7]. By disrupting the cellular and structural elements of the microvasculature, nonenzymatic glycation of collagen thickens the capillary basement membrane by altering the microvasculature’s cellular and structural elements. In diabetic microangiopathy, this is the primary morphological alteration [29,30].

In our study, while comparing diabetic neuropathy and cheiroarthropathy, 15 (34.9%) cheiroarthropathy patients had neuropathy compared to 14.9% without cheiroarthropathy, which has a statistically significant difference. We found that there was an increased incidence of diabetic neuropathy in subjects with cheiroarthropathy. Larkin et al. in the study on diabetes mellitus and diabetic cheiroarthropathy also showed a positive association between cheiroarthropathy and diabetic neuropathy [9].

On comparing diabetic retinopathy and diabetic cheiroarthropathy, 30 (35.7%) patients with diabetic cheiroarthropathy had diabetic retinopathy compared to 9.6% without, which has a statistically significant difference. We found an increased incidence of diabetic retinopathy in patients with diabetic cheiroarthropathy. Frost and Beischer had similar findings that showed an increased incidence of retinopathy in patients with diabetic cheiroarthropathy [10]. Similar results were obtained in a study done in Nigeria, which showed an increased prevalence of retinopathy in patients with limited joint mobility [14].

Comparing diabetic nephropathy and diabetic cheiroarthropathy, 26 (26.8%) patients with cheiroarthropathy had nephropathy compared to 13% without, which has a statistically significant difference. This was similar to a previous study done by Garg et al., who demonstrated an increased prevalence of microalbuminuria in a cross-sectional study of 357 subjects [13]. A 15-year prospective study of 37 individuals with type 1 DM found that those with microalbuminuria experienced a larger loss in joint mobility than those without it [16].

Limited joint mobility is difficult to treat. Sibbitt and Eaton found that corticosteroid injections into the palmar tendon sheath could reduce the contractures caused by restricted joint motion [31]. It is possible to alleviate the symptoms and indications by achieving better glycemic control. Some authors even assert that DCA could be completely reversed [32,33]. Despite the fact that many cross-sectional studies have not supported a link between LJM and good control of DM, certain studies have demonstrated an elevated risk of LJM in the context of higher hemoglobin A1c (HbA1c) levels [15]. Anti-inflammatory medications, joint and muscle stretching exercises, or physiotherapy are also useful to improve joint mobility in these patients.

Although there have not been any significant studies showing how better control of blood glucose affects LJM, there are few empirical evidence. Case studies have shown that hand alterations can be resolved with better DM control [32]. It has been hypothesized that stretching the toes and subtalar joint can lower the incidence of foot ulcers; however, this has not been verified.

## Conclusions

There is an increased prevalence of diabetic nephropathy, diabetic neuropathy, and diabetic retinopathy in patients with diabetic cheiroarthropathy. The presence of diabetic cheiroarthropathy hence gives us an idea about the increased microvascular complications of diabetes mellitus. The diagnosis of diabetic cheiroarthropathy is clinical and does not require laboratory-based investigations and can be used as a marker for the presence of diabetes-related microvascular complications in resource-poor settings. The presence of diabetic cheiroarthropathy hence warrants better control of the glycemic status of the patient to prevent further deterioration from diabetes-related complications.
